# Evaluating the presence of *aggregatibacter actinomycetemcomitans*, *porphyromonas gingivalis* levels in patient's self- ligating brackets

**DOI:** 10.6026/973206300213235

**Published:** 2025-09-30

**Authors:** Dhananjay Rathod, Anukrati Doneria, Subasish Behera, Soumyaranjan Nanda, Sheetal Acharya, Ananya Bhargava, Miral Mehta

**Affiliations:** 1 Department of Dentistry, Sheikh Bhikhari Medical College, Hazaribagh, Jharkhand, India; 2 Department of Dental Surgery,SN Medical college, Agra, Uttar Pradesh, India; 3 Department of Conservative Dentistry and Endodontics, Government Dental College and Hospital (SCB), Manglabag, Cuttack, Odisha, India; 4 Endodontics, Private Practitioner, Cuttack, Odisha, India; 5 Department of Periodontics and Implantology, Kalinga Institute of Dental Sciences KIIT (Deemed to be) University, Bhubaneswar, India; 6 Department of Dentistry, Ruxmaniben deepchand gardi medical college, Ujjain, Madhya Pradesh, India; 7 Department of Pediatric and Preventive Dentistry, Karnavati School of Dentistry, Karnavati University, Gandhinagar, Gujarat, India

**Keywords:** Self-ligating brackets, *aggregatibacter actinomycetemcomitans*, *porphyromonas gingivalis*, orthodontic microbiology, periodontal pathogens, RT-PCR

## Abstract

The impact of self-ligating brackets on periodontal health remains uncertain, particularly regarding colonization by key pathogens such as *aggregatibacter actinomycetemcomitans* and *porphyromonas gingivalis*. Therefore, it is of interest to evaluate microbial changes in 40 orthodontic patients fitted with self-ligating brackets. Subgingival plaque samples were collected at baseline, 1 month and 3 months and analyzed using real-time PCR. Results showed a significant rise in *P. gingivalis* and a moderate increase in *A. actinomycetemcomitans*, with the highest accumulation observed around posterior brackets. Thus, we show that self-ligating brackets may favor pathogen colonization, highlighting the importance of strict oral hygiene measures to reduce periodontal risks.

## Background:

Orthodontic treatment using fixed appliances significantly influences the oral microbiome due to the increased retention of plaque around brackets and wires, leading to microbial dysbiosis [1]. Self-ligating brackets (SLBs) have been introduced as an alternative to conventional ligating systems, with the claim that they provide better oral hygiene due to the absence of elastic ligatures, which are known to attract plaque and microorganisms [2]. However, the evidence regarding their effect on the levels of specific periodontal pathogens remains inconclusive. Among the key pathogens involved in periodontal disease, *aggregatibacter actinomycetemcomitans* (Aa) and *porphyromonas gingivalis* (Pg) are regarded as critical contributors to periodontal tissue destruction and bone resorption [3, 4]. These microorganisms are associated with aggressive periodontitis and chronic periodontal inflammation, respectively. Their ability to colonize subgingival sites and evade host immune mechanisms makes them particularly relevant in orthodontic settings, where plaque control is often compromised [5]. Studies have shown that the design of orthodontic brackets can significantly influence bacterial colonization. SLBs, while offering mechanical advantages like reduced treatment time and friction, may still present surfaces conducive to biofilm accumulation [6]. The literature is divided on whether SLBs reduce or exacerbate microbial presence when compared to conventional systems [7, 8]. While some investigations report lower bacterial adherence due to the smoother surface of SLBs, others suggest that the mechanical components of SLBs create niches favorable for bacterial retention [9]. Given the pathogenic role of *A. actinomycetemcomitans* and *P. gingivalis* and the rising popularity of SLBs in orthodontics, it becomes critical to investigate whether such appliances influence the colonization patterns of these microorganisms [9]. This study addresses this gap by using sensitive molecular techniques (RT-PCR) to quantify the presence of these pathogens in patients undergoing orthodontic treatment with self-ligating brackets, thereby contributing valuable data toward periodontal risk assessment during orthodontic therapy [10]. Molecular techniques such as real-time polymerase chain reaction (RT-PCR) have enhanced the accuracy of detecting periodontal pathogens, enabling quantitative assessment of specific species in clinical samples [10]. Therefore, it is of interest to report the colonization patterns of *A. actinomycetemcomitans* and *P. gingivalis* in patients treated with self-ligating brackets using RT-PCR analysis.

## Materials and Methods:

## Study design and population:

This prospective observational study was conducted on 40 orthodontic patients aged between 14 and 25 years who reported to the Department of Orthodontics and Dentofacial Orthopedics. All participants were fitted with self-ligating brackets (passive type) for fixed orthodontic therapy. Ethical clearance was obtained from the institutional ethical review board and informed consent was taken from all subjects or their guardians before study enrollment.

## Inclusion and exclusion criteria:

Participants with healthy systemic status, good baseline periodontal health and no recent antibiotic usage (within 3 months) were included. Exclusion criteria included individuals with systemic diseases, existing periodontal disease, ongoing anti-inflammatory or antibiotic therapy and smokers.

## Sample collection:

Subgingival plaque samples were collected from the mesiobuccal surface of first molars and central incisors using sterile Gracey curettes at three time intervals: baseline (prior to appliance placement), one month and three months after appliance bonding. Samples were transferred immediately into sterile Eppendorf tubes containing 1 mL of TE buffer and stored at -20°C until further analysis.

## DNA extraction and real-time PCR analysis:

Genomic DNA was extracted using a commercially available bacterial DNA isolation kit according to the manufacturer's instructions. The concentration and purity of DNA were verified using a NanoDrop spectrophotometer. Quantitative real-time polymerase chain reaction (qRT-PCR) was performed using species-specific primers targeting 16S rRNA genes of *aggregatibacter actinomycetemcomitans* and *porphyromonas gingivalis*. Amplification was carried out in a thermal cycler using SYBR Green chemistry. Standard curves for quantification were generated using serial dilutions of known bacterial DNA concentrations.

## Statistical analysis:

Data were entered in Microsoft Excel and analyzed using SPSS software version 25.0. Mean and standard deviation were calculated for the bacterial counts at different time points. Repeated measures ANOVA was used to assess within-group variations and post-hoc Bonferroni tests were applied to determine significant differences between intervals. A p-value of <0.05 was considered statistically significant.

## Results:

A total of 40 patients (20 males and 20 females) completed the study. The mean age of participants was 19.2 ± 3.6 years. The levels of *aggregatibacter actinomycetemcomitans* and *porphyromonas gingivalis* were evaluated at baseline, 1 month and 3 months following the placement of self-ligating brackets. There was a progressive increase in the bacterial count of both species over time. *A. actinomycetemcomitans* showed a statistically significant increase from baseline to 1 month, with a slight decline by the third month. *P. gingivalis* levels demonstrated a consistent and significant rise across all time points. Table 1 (see PDF) shows the mean bacterial counts (in CFU/mL) for *A. actinomycetemcomitans* at three different time intervals. There was a significant rise from 2.1x10^4^ ± 0.6 at baseline to 4.6x10^4^ ± 0.8 at 1 month, followed by a slight decline to 4.2x10^4^ ± 0.9 at 3 months (p<0.05). Table 2 (see PDF) displays the progression of *P. gingivalis* levels over time. The bacterial count increased from 1.7x10^4^ ± 0.5 at baseline to 3.9x10^4^ ± 0.6 at 1 month and further to 5.9x10^4^ ± 0.7 at 3 months (p<0.01). Table 3 (see PDF) compares the mean bacterial load based on arch location (maxilla vs. mandible) at 3 months. Both pathogens exhibited significantly higher concentrations in the mandibular arch (p<0.05), with *P. gingivalis* showing the highest colonization. Table 4 (see PDF) presents inter-group comparisons based on oral hygiene compliance (assessed via patient-reported frequency of brushing). Patients with poor oral hygiene (≤1 brushing/day) exhibited significantly higher bacterial counts for both organisms compared to those brushing twice daily (p<0.01). As shown in Tables 1-4 (see PDF), the presence of self-ligating brackets led to increased colonization of periodontal pathogens, with hygiene practices and anatomical location influencing bacterial levels.

## Discussion:

This study aimed to assess the colonization patterns of *aggregatibacter actinomycetemcomitans* and *porphyromonas gingivalis* in patients undergoing orthodontic treatment with self-ligating brackets over a three-month period. The findings revealed a notable increase in the levels of both periodontal pathogens, particularly *P. gingivalis*, which showed a significant rise from baseline to the third month. These results align with previous research indicating that fixed orthodontic appliances influence the oral microbiota by creating new ecological niches for bacterial colonization [1, 2]. Although self-ligating brackets are marketed as being more hygienic due to the absence of elastomeric ligatures, their structure may still provide sites for microbial retention, especially in the posterior regions where plaque removal is more difficult [3, 4]. Our study supports the observations made by van Gastel *et al.*, who found similar increases in pathogenic bacterial loads around fixed appliances, regardless of ligation method [5]. Moreover, *P. gingivalis* demonstrated greater colonization than *A. actinomycetemcomitans*, likely due to its anaerobic preferences and ability to thrive in mature biofilms found in periodontal pockets [6, 7]. The use of real-time PCR provided an accurate and quantitative analysis of microbial levels, allowing for more sensitive detection compared to culture methods [8]. Studies by Fine *et al.* and How *et al.* emphasize the clinical relevance of quantifying these pathogens due to their potent virulence factors such as leukotoxin and gingipains, which directly contribute to tissue destruction and immune evasion [9, 10]. The increasing trend of these bacteria over time, especially in the mandibular arch, suggests the influence of anatomical factors, such as limited access for mechanical cleaning and saliva pooling, which favors bacterial growth [11]. Interestingly, our results demonstrated that oral hygiene practices had a significant effect on microbial load. Patients who maintained better brushing compliance exhibited reduced levels of both pathogens after 3 months. This emphasizes the need for rigorous hygiene protocols in patients undergoing fixed orthodontic therapy, even when using self-ligating brackets [12]. According to studies by Buck *et al.* and Turkkahraman *et al.* oral hygiene practices directly correlate with plaque accumulation and gingival inflammation in orthodontic patients [13, 14]. The marginal decline in *A. actinomycetemcomitans* after the first month may be due to host immune adaptation or the competitive suppression by other microbial species, including *P. gingivalis*, known for its dominance in mature subgingival biofilms [15]. The insertion of fixed orthodontic appliances induces an increase in plaque formation associated with a qualitative bacterial shift from aerobic to anaerobic micro flora that endangers the integrity of hard and soft tissues [16]. MBT brackets were associated with higher bacterial colonization compared to self-ligating brackets, indicating that bracket design influences microbial accumulation and may impact periodontal health during orthodontic treatment [17].

## Conclusion:

In summary, while self-ligating brackets offer certain clinical advantages, they do not eliminate the risk of increased colonization by periodontal pathogens. Thus, strict oral hygiene instruction, regular periodontal monitoring and possibly adjunctive antimicrobial strategies should be considered for patients undergoing treatment with such appliances.
